# Accelerating writing engagement: a design framework for operationalizing student engagement in writing instruction

**DOI:** 10.3389/fpsyg.2026.1461683

**Published:** 2026-02-26

**Authors:** Jonathan Marine, Seth A. Parsons, Samantha T. Ives, Paul Kessler Rogers

**Affiliations:** 1 Department of English & Creative Writing, Stephen F. Austin State University, Nacogdoches, TX, United States; 2 George Mason University, Sturtevant Center For Literacy, Fairfax, VA, United States; 3 University of California, Santa Barbara, Gevirtz School of Education, Santa Barbara, CA, United States

**Keywords:** accelerated learning theory, composition theory, engagement theory, writing engagement, writing instruction

## Abstract

In this conceptual article, we argue that Accelerated Learning Theory (ALT) provides a pedagogical design framework for operationalizing multidimensional writing engagement through instructional sequencing, task design, and participation structures. We synthesize ALT with multidimensional engagement theory to address a persistent theory-to-practice challenge: although engagement is strongly associated with positive learning outcomes, it remains difficult to translate engagement constructs into actionable classroom design decisions, particularly in the realm of writing instruction. We articulate a set of ALT-informed design levers (e.g., multimodal access, participation structures, learning climate, relevance, and iterative feedback) that support affective, behavioral, cognitive, and social engagement in writing. We conclude with illustrative classroom practices and an example scenario that demonstrate how the framework can guide teachers’ decisions about writing instruction and outline directions for future empirical research.

## Introduction

Educators remain in continuous pursuit of effective teaching methods because learning is a sophisticated and nuanced activity which continues to evolve in schools and societies the world over. As theories of learning advance, they provide potential opportunities to support effective instruction through illuminating effective teaching and learning strategies and practices. Two prominent theories that have garnered attention in educational psychology are accelerated learning theory (ALT) and engagement theory. ALT emphasizes the creation of dynamic and immersive learning experiences that maximize student understanding, retention, and satisfaction (McKeon, 1995; Safitri and Umamah, 2019), while engagement theory focuses on fostering students’ active involvement, interest, and motivation (Fredricks et al., 2004; Christenson and Reschly, 2012). While these theories emerge from distinct perspectives, we argue that they converge around principles fundamental to effective learning and that ALT can serve as a means of addressing a persistent theory-to-practice gap in engagement research.

Despite the robust theoretical foundations and growing empirical evidence demonstrating that student engagement is associated with positive academic outcomes (Fredricks et al., 2004; Reeve et al., 2012), operationalizing engagement in day-to-day instructional practice remains a persistent challenge (Reeve and Shin, 2020; Christenson and Reschly, 2012; Trowler, 2010). Engagement is a multidimensional and dynamic construct, encompassing affective, behavioral, cognitive, and social dimensions that do not emerge uniformly or simultaneously during learning activities. Much of the engagement literature has therefore focused on construct validation and outcome prediction, demonstrating that engagement matters, while offering more limited guidance regarding how teachers might intentionally design instruction to foster engagement under real classroom conditions. As a result, engagement-oriented recommendations often remain at the level of general principles, such as providing choice, authenticity, or collaboration, leaving unanswered more specific questions about instructional sequencing, degrees of structure, and task design. This theory-to-practice gap is particularly salient in writing instruction, where tasks are complex, time-intensive, and highly sensitive to instructional design decisions.

To this end, in this paper we argue that Accelerated Learning Theory (ALT) offers a productive pedagogical design lens for addressing the challenge of operationalizing engagement with writing. Rather than redefining engagement, ALT foregrounds instructional conditions, sequencing, and participation structures that shape how quickly and robustly engagement emerges during learning activities. ALT emphasizes early success, multimodal access, supportive learning environments, and iterative feedback as mechanisms for accelerating learners’ entry into productive engagement and sustaining it over time (Boyd, 2004; Brookfield, 2003; Fors et al., 2013; Ganiron, 2013; Hyland and Hyland, 2006; Patchan et al., 2016). When paired with engagement theory’s multidimensional conceptualization, ALT provides a means of translating engagement from an abstract construct into a set of intentional instructional design decisions. In this way, engagement theory specifies the target states of learning, while ALT specifies the design logic through which those states can be cultivated in writing instruction.

An area where the intersection of these theories holds particular promise is in the realm of writing instruction. Writing is not only a fundamental academic skill but also a means of expression and communication essential for success across all aspects of modern life (Bazerman, 2009). However, engaging students in the writing process and fostering their development as proficient writers poses significant challenges for educators (Graham and Perin, 2007). By leveraging principles from ALT to drive engagement with writing, educators can design and implement writing instruction activities that are effective in accelerating writing skill development and that engage student learners with writing and the writing process.

With this goal, the purpose of this article is to propose an instructional design framework that integrates ALT and writing engagement to address persistent challenges in operationalizing engagement in writing instruction. We examine how ALT can serve as a pedagogical mechanism for enacting multidimensional writing engagement by guiding instructional sequencing, task design, and classroom participation structures. To do so, we first review the key principles and theoretical foundations of ALT and engagement theory. We then elaborate the construct of writing engagement and examine how principles derived from ALT can be leveraged to operationalize engagement in instructional practice. Next, we illustrate how this integrated framework can inform classroom decision-making through concrete examples of writing instruction, highlighting implications for teaching and future research. Finally, we consider the implications of this framework for classroom practice, acknowledge its conceptual limitations, and outline directions for future empirical research.

## Accelerated learning theory: a comparative analysis

Accelerated Learning Theory (ALT) is a pedagogical approach concerned less with identifying new instructional practices than with organizing *how* learning experiences are designed, sequenced, and supported to promote effective learning (Hoffman et al., 2010; McKeon, 1995). Although ALT draws on well-established traditions such as constructivism, sociocultural theory, and experiential learning, its distinctive contribution lies in foregrounding instructional conditions, such as emotional safety, early success, multimodal access, and iterative feedback, and the ways these conditions shape learners’ participation over time (Brookfield, 2003; Fors et al., 2013). In this sense, ALT functions less as a learning theory and more as a design-oriented framework that emphasizes how instructional decisions can accelerate learners’ entry into productive engagement and sustain it throughout complex learning tasks.

Importantly, many instructional practices associated with ALT such as collaboration, authenticity, student choice, and active learning are already well-theorized within engagement research and literacy scholarship (Fredricks et al., 2004; Guthrie and Humenick, 2004; Flowerday and Schraw, 2003; Parsons et al., 2015, 2018). ALT claims neither ownership nor origination of these practices, nor does it suggest that engagement depends upon ALT for its realization. Rather, ALT *reframes* these practices by emphasizing their sequencing, integration, and cumulative effects on learners’ engagement. From this perspective, ALT offers value not by introducing new pedagogical moves, but by providing a *coherent design logic* for how familiar practices can be combined and enacted to support learning efficiently and sustainably. ALT emphasizes the design of learning environments that promote active participation through experiential, collaborative, and contextually meaningful activity (Ganiron, 2013; Wild, 2007). Its contribution lies not in these practices themselves, but in specifying how they are sequenced, supported, and integrated over time to shape learners’ engagement and accelerate learning (Fisher et al., 2021).

The origins of ALT can be traced back to pioneering educational theorists such as Dewey, Piaget, and Vygotsky, whose work laid the groundwork for many contemporary approaches to teaching and learning (Maume and Matthews, 2000). Both Dewey (1938) emphasis on ‘learning by doing’ and Piaget (1957) constructivist theory of cognitive development have profoundly influenced the conceptualization of ALT, emphasizing the importance of experiential learning and active engagement (Watanabe, 2017). Similarly, Vygotsky’s (1978) sociocultural theory underscores the role of social interaction and collaborative learning in cognitive development, providing further insights into the principles of ALT (Schmidt, 2008). These theoretical roots situate ALT within long-standing educational traditions, while clarifying its contribution as a framework for translating those traditions into concrete instructional design principles.

From a theoretical standpoint, ALT encompasses a diverse array of different frameworks and models that all seek to illuminate the different cognitive and psychological processes underlying learning. One prominent model within the ALT framework is Bloom’s Taxonomy, which delineates hierarchical levels of cognitive complexity, ranging from basic recall to critical thinking and creativity (Radmehr and Drake, 2019). Within an ALT framework, such models are used not as static hierarchies but as guides for designing instructional sequences that move learners toward increasingly complex forms of cognitive engagement. By scaffolding instruction to progressively challenge learners at higher levels of cognitive demand, educators can promote deeper learning and skill acquisition (Serdyukov, 2008).

ALT also intersects with contemporary research on motivation, self-regulation, and metacognition, offering valuable insights into the factors that influence learning behavior and academic performance (Asri and Suhaili, 2022; Purnami et al., 2018). As Latino et al. (2020) report, minority students “who participated in accelerated learning had significantly higher GPAs on average than those who did not” (p. 456). Self-determination theory, for instance, emphasizes the role of autonomy, competence, and relatedness in fostering intrinsic motivation and engagement (Deci and Ryan, 2012). Within ALT, these motivational and metacognitive constructs are not treated as learner traits alone, but as outcomes shaped by instructional design features such as task structure, feedback timing, and opportunities for supported practice.

Practical applications of ALT encompass a wide range of instructional strategies and techniques designed to optimize learning outcomes across diverse educational contexts. These include inquiry-based learning, project-based learning, flipped classrooms, collaborative learning communities, and digital technologies (Ganiron, 2013; Hoffman et al., 2010). Overall, ALT represents a multifaceted approach to education that emphasizes personalized instruction, active engagement, and meaningful learning experiences. Rooted in cognitive, psychological, and sociocultural perspectives, ALT offers a holistic framework for optimizing the learning process and promoting student success and educators can harness ALT’s design principles to support sustained engagement and learning across diverse educational contexts. We note that portions of the ALT literature remain practice-facing and uneven in theoretical depth, underscoring the need for continued empirical and conceptual work. It is for this reason that coalescing ALT principles with engagement theory represents a particularly productive opportunity.

## Writing engagement: concepts and practices

The construct of engagement has received increasing attention since Fredricks et al. (2004) pivotal article in *Review of Educational Research*. Recently, for example, the second edition of the *Handbook of Research on Student Engagement* (Reschly and Christenson, 2022) was released, and research has demonstrated that engagement is associated with positive student outcomes, including achievement (Lei et al., 2018). Building upon other researchers such as Fredricks et al. (2004) and Alexander (2018), we conceptualize engagement as a multidimensional construct that includes affective, behavioral, cognitive, and social dimensions (Rogers et al., 2022). Affective engagement (also sometimes called emotional engagement) includes students’ interests, enthusiasm, and enjoyment. Behavioral engagement refers to students’ active participation, often operationalized as time-on-task. Cognitive engagement involves metacognition and strategic thinking and action. Social engagement is collaborating with others to complete a task.

Scholars have applied a multidimensional conceptualization of engagement to various disciplines, including science (Hampden-Thompson and Bennett, 2013), math (Liu et al., 2018), reading (Guthrie et al., 2012), and more. In our work, we apply it to writing (Parsons et al., 2023; Rogers et al., 2022). Students are affectively engaged in writing when they are enthusiastic about writing and enjoy the process. They are behaviorally engaged as they participate in the act of writing, including the many components of the writing process (Ives et al., 2022). Students are cognitively engaged when they are thoughtful and metacognitive in their writing processes. And students are socially engaged in writing when they work with others to brainstorm, draft, revise, edit, or share their writing.

This multidimensional conceptualization of writing engagement provides a framework for identifying the forms of engagement teachers seek to support during writing instruction (Fredricks et al., 2004; Rogers et al., 2022). While engagement theory clarifies what affective, behavioral, cognitive, and social engagement look like in writing (Alexander, 2018; Ives et al., 2022), it offers more limited guidance regarding how teachers might intentionally design learning environments and tasks to foster these dimensions in concert (Trowler, 2010). In particular, engagement theory does not fully specify how instructional decisions related to sequencing, task structure, degrees of choice, and participation formats influence the timing and durability of students’ engagement during writing (Flowerday and Schraw, 2003; Guthrie et al., 2012). Addressing these design challenges requires a complementary pedagogical lens capable of translating engagement constructs into instructional action. In the following section, we examine how principles derived from ALT provide such a lens, offering concrete guidance for operationalizing writing engagement through intentional instructional design.

## Intersections of ALT and writing engagement

In this section, we move beyond identifying conceptual alignment between ALT and writing engagement to examine how ALT functions as an instructional design lens for operationalizing engagement in writing instruction. Specifically, ALT offers guidance for how teachers make decisions about task structure, participation formats, sequencing, and learning conditions in ways that support affective, behavioral, cognitive, and social dimensions of writing engagement. The following design levers illustrate how principles derived from ALT can be enacted to accelerate and sustain students’ engagement with writing.

## Multisensory and multimodal access as a design lever

A central principle of ALT is multisensory engagement, which emphasizes designing learning experiences that engage multiple sensory and representational modalities (Boyd, 2004; Fors et al., 2013). In writing instruction, this principle supports engagement by expanding how students enter, process, and express ideas through writing-related activity. Writing is not solely a cognitive act; it is also social, affective, and embodied (Baart, 2002; Perl, 2004; Zabihi, 2018). Designing writing tasks that incorporate discussion, visual representation, oral rehearsal, and collaborative meaning-making allows students multiple points of entry into the writing process. Socially mediated writing activities, in particular, activate both social and affective engagement, supporting learners as they co-construct meaning and sustain motivation (Zhang, 2021). Through ALT, multisensory engagement becomes a deliberate design choice that lowers barriers to participation and accelerates students’ engagement with writing.

## Active participation and participation structures

ALT foregrounds active participation as a core condition for learning, emphasizing inquiry, collaboration, and learner agency (Mohamed, 2012). Rather than treating participation as an outcome, ALT positions it as something that must be intentionally designed through instructional structures. In writing instruction, participation structures such as peer discussion, collaborative drafting, and small-group workshops shape how students engage behaviorally and socially with writing tasks (Bean and Melzer, 2021; Freedman et al., 2005). ALT contributes by encouraging teachers to design participation formats that move students from supported interaction toward increasingly independent writing activity. In this way, ALT supports the coordination of behavioral and social engagement through explicit instructional design.

## Learning climate and affective conditions

Another design emphasis within ALT is the creation of positive learning environments that support emotional safety, persistence, and risk-taking (Safitri and Umamah, 2019). Writing instruction is particularly sensitive to affective conditions, as students often associate writing with vulnerability, evaluation, and fear of failure (Graham and Perin, 2007; Rogers et al., 2022; Troia et al., 2012). ALT highlights the role of instructional climate in shaping affective engagement by encouraging teachers to normalize revision, celebrate effort, and frame mistakes as opportunities for learning. Empirical work supports the importance of these conditions, with evidence that interventions targeting wellbeing, resilience, and emotional regulation positively influence writing achievement (Rad and Jafarpour, 2023). Through ALT, affective engagement is supported not only through encouragement, but through intentional design of classroom norms and feedback practices.

## Relevance, interest, and task design

ALT also emphasizes relevance as a driver of engagement, suggesting that learning accelerates when tasks connect to learners’ interests, experiences, and values (Wilkins et al., 2010). In writing instruction, relevance becomes a task-design decision: teachers determine which elements of an assignment are fixed and which are open to student choice. Research suggests that writing about personally meaningful topics can enhance motivation and self-worth (Crocker et al., 2008), supporting affective and cognitive engagement. ALT contributes by framing relevance not as an add-on, but as a strategic design lever that influences how quickly students invest effort in writing tasks and sustain engagement over time.

## Variety, feedback, and iterative learning

ALT encourages instructional variety and iterative feedback as means of maintaining engagement and supporting learning progression (Marques, 2012). In writing instruction, variety in genres, modes, and composing processes allows students to engage with writing in ways that align with their strengths while developing new competencies. Feedback and revision play a central role in this process, supporting cognitive engagement through reflection and strategic revision (Hyland and Hyland, 2006; MacArthur, 2012; Patchan et al., 2016). ALT frames feedback as part of an ongoing design cycle rather than a terminal evaluation, emphasizing timing and frequency as critical factors in sustaining engagement.

## Intrinsic motivation and autonomy-supportive design

Finally, ALT aligns with research on intrinsic motivation by emphasizing autonomy, competence, and purpose as outcomes of instructional design (Asri and Suhaili, 2022; Camacho et al., 2021; Troia et al., 2012). In writing instruction, autonomy-supportive design includes opportunities for creative self-expression, meaningful choice, and authentic audiences. Such conditions have been shown to support the development of intrinsically motivated writers who engage deeply across genres (Wright et al., 2020). ALT contributes by emphasizing how these motivational conditions are cultivated through task structure, sequencing, and participation opportunities, rather than assumed as learner dispositions.

Taken together, these design levers illustrate how ALT operationalizes writing engagement by guiding instructional decisions that shape when, how, and to what extent students engage affectively, behaviorally, cognitively, and socially with writing. Rather than introducing new pedagogical practices, ALT provides a coherent framework for organizing and enacting engagement-supportive instruction in writing classrooms.

## Effective classroom practices

In this section, we illustrate how the design principles articulated above can be enacted in classroom practice. Focusing on the roles of the teacher and the task, we highlight how instructional decisions related to learning climate, participation structures, task design, and sequencing can operationalize writing engagement in everyday instructional contexts.

When considering instructional practices that embody the characteristics of accelerated learning theory and writing engagement, we focus on two vital aspects of classroom practice: the teacher and the task. Scholars have argued that the teacher is the most important in-school factor in students’ learning (Darling-Hammond, 2000). Teachers wield a lot of power in the classroom. A favorite education quote of ours illustrates this power:

I have come to the frightening conclusion that I am the decisive element. It is my personal approach that creates the climate. It is my daily mood that makes the weather. I possess tremendous power to make life miserable or joyous. I can be a tool of torture or an instrument of inspiration, I can humiliate or humor, hurt or heal. In all situations, it is my response that decides whether a crisis is escalated or de-escalated, and a person is humanized or de-humanized. (Ginott, 1972, p. 15).

Teachers determine the classroom environment in the procedures, norms, expectations, interactions, and culture established. Within that environment, they provide instruction and create and assign academic tasks, and the tasks teachers assign dictates how students participate in school (Parsons and Scales, 2013). From a social constructivist perspective, what students do determines what they learn. Together, these aspects of the classroom greatly influence student learning and student engagement. Therefore, we encourage educators to carefully attend to the classroom environment, their interactions with students, and the tasks they assign.

### The teacher

Teachers set the tone for what happens in their classrooms and their primary role is to ensure that students learn the knowledge and skills essential to various content areas. For students to excel in their learning, they must be engaged. When teachers understand ALT and are aware of student engagement principles, they are better equipped to help students succeed. For example, such teachers can create a safe and respectful learning environment where creativity and exploration is encouraged. Failure is positioned as a learning opportunity rather than something that is punished. Collaboration is embedded within learning processes where students and teachers engage in dialogue as they ask questions, apply their learning, solve problems, wrestle with ideas, unpack content, and so on. From an ALT perspective, these teacher actions function as design levers that shape affective, behavioral, cognitive, and social engagement during writing instruction.

#### Accelerating affective writing engagement

As previously described, affective engagement involves students’ interests and emotions as they participate in school. To tend to affective engagement, teachers can use what they know about ALT and their students to create an environment in which students feel comfortable, encouraged, and excited. In writing, this can include opportunities for students to write about topics in which they are personally invested. Allowing students to demonstrate their expertise about topics of interest through writing is a way for students to both practice their writing skills and enjoy the process.

#### Accelerating behavioral writing engagement

Behavioral engagement is the most apparent type of engagement and is something that teachers monitor throughout the school day. This is done by ensuring students are focused on learning and working on assignments. Something that teachers can do to support students’ behavioral writing engagement is to provide time in class for students to participate in various forms of writing. Because writing is time-intensive, teachers can end up assigning it as homework. However, there is also value in providing time and space for students to go through the entire writing process where teachers can monitor and support students’ behavioral engagement in each part of the process. Central to ALT is active participation with teacher guidance feedback, which provides students with experiential learning in combination with expert guidance from the teacher.

#### Accelerating cognitive writing engagement

Cognitive engagement, or strategic thinking about the task at hand, is the third type of engagement teachers can foster in their classrooms. Writing is an immensely complex skill. Luckily, teachers are prepared to teach their students various strategies to expand and strengthen their writing. A simple way to teach students writing strategies is through the gradual release of responsibility model (Webb et al., 2019). This model includes four steps (Fisher and Frey, 2021). First, the teacher models the writing strategy for their class. Next, through guided instruction, teachers and their students work together to practice using the strategy. In the third step, which also connects to social engagement, students work together practicing their targeted writing strategy. Finally, in the fourth step students work on their writing strategy independently and demonstrate their understanding.

#### Accelerating social writing engagement

The fourth type of engagement is social engagement in which students interact with their teachers and peers. Writing lends itself well to environments in which teachers can foster social engagement because a core purpose of writing is communicating with others. Teachers should create environments in their classrooms in which students feel comfortable to both share writing with their peers and respond to their peers’ writing supportively. Teachers need to model these types of exchanges in front of their students so that they can learn how to collaborate during the writing process.

### The task

Education scholars have long considered the academic task as a central feature of teaching because it is so closely related to student learning (Blumenfeld et al., 1987; Doyle, 1983; Parsons and Scales, 2013). The task, or the activity that students are assigned, establishes what students are supposed to do—how they will participate in the activity. And we know from early and current theorists that *doing* facilitates learning (Dewey, 1938; Skulmowski, 2024). Applied specifically to writing, the writing assignment is central to how students engage with writing. The format, purpose, audience, length, topic, duration, etc. are all selected by the teacher, who can also open some of the selecting to students. Therefore, teachers can optimally engage students in writing and maximize student learning by assigning well-designed tasks.

#### Relevance and authenticity

In line with ALT, engaging tasks compel students to apply what they are learning in ways that are relevant to their lives (Wilkins et al., 2010). Both engagement and learning is heightened when students can relate to the material and can see its relevance in their lives. Aligning content with students’ lives also corresponds with culturally responsive pedagogy, instruction that is mindful of and responsive to students’ cultures (Turner et al., 2021). A closely related factor is authenticity. Authentic tasks mimic real-world activity. Pearson et al. (2007) clearly present the case for authenticity:

The argument underlying the promotion of authenticity is that too many school tasks are unauthentic, unrealistic, and, by implication, not useful for engaging in real-world literacy activities; that is, instead of teaching kids how to “do school,” we should be teaching them how to “do life.” (p. 36).

Authentic tasks give students a genuine purpose for engaging in the activity. Writing in ways that extend beyond “writing for school” can heighten students’ engagement. Many forms of writing that occur in the real world can easily be brought into the curriculum. Book reviews, poems, letters to the editor, memoirs, research articles, historical accounts, short stories, and on and on are all authentic types of writing that could be incorporated into instructional activities. Gambrell et al. (2011), for example, studied the role of authenticity in a project where students wrote to pen pals. The real-world task with a real-world audience made this project authentic to students. They found that increased authenticity led to increased motivation and critical thinking for the elementary students. Having a real-world audience is another easy way to increase the authenticity of writing tasks.

#### Student choice

Student choice is another aspect of instruction that aligns with ALT (Wilkins et al., 2010) and is associated with increased engagement (Ives et al., 2021). When we give students choice, we provide them self-determination in their writing, which supports them in taking ownership of the task and product. When students have meaningful choices in tasks, they have autonomy within the class. That is, they have a level of control of their participation. The opposite situation is a controlled environment, where all decisions are made by someone else—typically the teacher in schools. Controlling environments do not position students as individuals who have specific needs, backgrounds, and interests. Controlling environments are antithetical to ALT and thwart student engagement (Ives et al., 2021). In designing writing assignments, we recommend that teachers thoughtfully consider who decides on the topic, the format, the audience, the content, and the process. The teacher can control the degree of student choice across all components. Sometimes students may choose the topic of their research, for example, while the teacher determines the format (how-to manual) and the audience (the other third grade class). But who chooses these aspects of the writing can vary based upon the assignment. Thus, teachers have the opportunity to give students choice on different aspects of writing tasks based upon the purposes of specific activities.

#### Open-ended tasks

ALT and research on student engagement support the use of open-ended writing tasks (Mohamed, 2012; Parsons et al., 2018). Open-ended tasks are less structured activities in which students work collaboratively and strategically to solve problems or accomplish a goal. Open-ended tasks are guided by an end product, which students work toward over time. This sustained learning aimed at coming to a solution or creating a product is aligned with ALT (Wild, 2007) and associated with student engagement (Guthrie and Humenick, 2004; Pressley, 2006). It is important to note that open-ended tasks are best for the application phase of learning—where students are applying what they learned to strengthen understanding. When students are learning new content or developing new skills, more structured forms of instruction are more appropriate (Skulmowski, 2024; Webb et al., 2019). However, applying and using their new knowledge in open-ended tasks can deepen students’ understanding and promote engagement after more direct forms of instruction present and explain new material (Kokotsaki et al., 2016). Open-ended tasks are a great place to infuse multisensory and multimodal instruction, which allow students to access and use knowledge in different ways, further solidifying learning.

## Classroom scenario

The following classroom scenario is intended as an illustrative example, drawn from our work in classrooms, of how ALT-aligned design principles can be enacted in writing instruction. Let us take a look at Mrs. Rucker’s fifth-grade class where her students are engaged in an open-ended writing task that aligns with ALT. The class is working on an ancient civilizations project. After brief overviews of each of the ancient civilizations over the last several weeks, small groups of students are assigned an ancient civilization to “represent.” In the instructional context, a time traveler is visiting each ancient civilization and the groups are responsible for teaching the time traveler about their civilization, its culture, its advancements, as well as its strengths and weaknesses as a society. They must write a speech that is accompanied by a multimodal presentation that includes visuals and dramatization. To accomplish this task, students collaborate in their groups for 3 weeks, conducting research, writing, designing, and creating.

This open-ended task is highly engaging for students. They are collaborating with peers and making choices as they participate in authentic tasks (writing a speech and presenting content to others). The assignment has space for creativity and requires multisensory and multimodal engagement with content. Throughout students’ work on the project, Mrs. Rucker pulls small groups for explicit teaching of skills, strategies, and content students need to work on their projects, and students who need more support receive it. Student writing is central in this project because they must present information in a clear, comprehensive (yet succinct) way, but they are motivated to engage in the writing because the open-ended project gives them autonomy, allows them to make the content relevant to their lives through multiple forms of presentation, and provides them support and guidance from the teacher (Parsons et al., 2018).

## Implications for practice and future research

Writing engagement is a vital aspect of effective literacy instruction, fostering students’ development as confident and proficient writers (Ives et al., 2022; Parsons et al., 2023). The framework advanced in this article suggests that cultivating robust engagement in writing requires more than identifying desirable engagement outcomes; it requires intentional instructional design. By drawing on principles from ALT and engagement theory in tandem, educators can move beyond general engagement-oriented recommendations toward more deliberate decisions about learning conditions, task structure, participation formats, and instructional sequencing.

At the core of this framework is the integration of ALT’s design logic with engagement theory’s multidimensional conceptualization of writing engagement, encompassing affective, behavioral, cognitive, and social dimensions (Rogers et al., 2022). Engagement theory clarifies what engaged writing looks like, while ALT provides guidance for how instructional environments and tasks can be designed to support these dimensions in concert. For practitioners, this means attending not only to whether students are engaged, but to how engagement is fostered through choices about task openness, multimodal access, feedback timing, and opportunities for supported participation. Such design-oriented thinking offers teachers a practical means of enacting engagement theory in the context of complex, time-intensive writing instruction.

From a theoretical perspective, this framework contributes to ongoing efforts to bridge the gap between engagement as a conceptual construct and engagement as an instructional outcome. Theories of social and human behavior have long emphasized either prediction or understanding (Dubin, 1978). Engagement research has been particularly effective in predicting academic outcomes and documenting the importance of engagement, while instructional frameworks such as ALT emphasize understanding the conditions under which learning occurs. By synthesizing these perspectives, the present framework seeks to advance understanding of how and why students engage in writing, rather than merely whether engagement is present.

## Limitations and directions for future research

Several limitations of this conceptual framework warrant consideration. First, the framework has not been empirically tested as an integrated model. Although both engagement theory and ALT are supported by extensive existing research, future studies are needed to examine how ALT-informed instructional designs specifically influence dimensions of writing engagement and writing performance. Second, the framework may not generalize uniformly across grade levels, instructional contexts, or writing genres, given the varied demands of writing tasks and learning environments. Third, disentangling the effects of ALT-aligned design from related factors such as teacher expertise, curricular constraints, and institutional context remains a methodological challenge.

Future research could address these limitations through empirical studies that explicitly operationalize ALT design principles in writing instruction. Mediation models may be useful for examining how instructional design features influence writing performance through dimensions of engagement. Longitudinal research designs could further illuminate the cyclical and developmental relationships between instructional conditions, engagement, and writing outcomes over time. Design-based research may also offer a productive approach for refining and testing ALT-informed engagement practices in authentic classroom settings.

## Conclusion

The exploration of ALT and writing engagement highlights their complementary roles in advancing writing instruction. Engagement theory provides a robust conceptualization of the dimensions of students’ involvement in writing, while ALT offers a pedagogical design lens for operationalizing those dimensions through instructional decision-making. By integrating these perspectives, educators can design writing instruction that intentionally supports affective, behavioral, cognitive, and social engagement across diverse contexts. This framework advances understanding of how engagement can be cultivated through instructional design and points toward productive avenues for future research. Continued empirical investigation of ALT-informed writing instruction holds promise for refining engagement theory, strengthening pedagogical practice, and improving student outcomes in writing.
